# Empowering nurses through inclusive leadership to promote research capacity building: A James Lind Alliance priority setting Partnership in Community Nursing

**DOI:** 10.1111/jan.15342

**Published:** 2022-06-28

**Authors:** Catherine Henshall, Louise Jones, Claire Armitage, Lee Tomlinson

**Affiliations:** ^1^ Faculty of Health and Life Sciences Oxford Brookes University Oxford UK; ^2^ Northumbria Healthcare NHS Foundation Trust Newcastle upon Tyne UK; ^3^ Directorate of Mental Health Partnership NHS Trust Leicestershire UK; ^4^ Kent Community Health NHS Foundation Trust London UK

**Keywords:** community care, leadership, nursing, nursing research, professional development, research in practice

## Abstract

**Aims:**

This paper focuses on the benefits of inclusive leadership when undertaking a priority setting partnership in community nursing, through providing a collaborative and committed nurse‐led forum for initiating impactful changes, identifying evidence uncertainties and driving research capacity‐building initiatives.

**Design:**

This is a Discussion paper. The project was undertaken between 2020 and 2021.

**Data sources:**

This paper is based on shared reflections as 70@70 Senior Nurse Research Leaders and is supported by literature and theory. It draws on issues relating to collective leadership, stakeholder engagement, diversity, inclusivity and COVID‐19.

**Implications for nursing:**

The James Lind Alliance Priority Setting Partnership catalysed the development of a rigorous evidence‐base in community nursing. The collaborative opportunities, networks and connections developed with patients, carers, nursing leaders, policy makers and healthcare colleagues raised the profile of community nursing research. This will benefit nursing research, practice, education and patients in receipt of community nursing care. Collective buy in from national leaders in policy, education, funding and commissioning has secured a commitment that the evidence uncertainties will be funded.

**Conclusion:**

Four key learnings emerged: collective leadership can ensure learning is embedded and sustained; developing an engaged stakeholder community to promote community nursing research is essential; a diverse membership ensures inclusivity and representation; and insights into the impact of COVID‐19 aid progress. The process increased research engagement and created capacity and capability‐building initiatives. This will help community nurses feel empowered to lead changes to practice. Sustained engagement and commitment are required to integrate research priorities into community nursing research, education and practice and to drive forward changes to commissioning and service delivery.

**Impact:**

The study promoted research capacity building through inclusive leadership. This can increase community nurses' research engagement and career development and patient care quality and safety; this can incentivize funders and policy makers to prioritize community nursing research.

## 1 INTRODUCTION

Globally, patients who participate in a research report greater satisfaction with care and improved clinical outcomes (Ozdemir et al., 2015). Similarly, research‐active healthcare organizations have lower mortality rates and higher quality care outcomes, highlighting the positive effects of healthcare research at an international level (Royal College of Physicians, 2020).

Nurses are the largest group of healthcare professionals worldwide and are continually engaged in innovative practice (Hughes, 2006), providing more patient‐facing care than other healthcare professionals. Because of their close proximity to patients, nurses are able to rapidly adapt and respond to international public health challenges adeptly and with expertise (Hughes, 2006); the recent COVID‐19 pandemic has illustrated this. This places nurses in a prime position to contribute to, and shape, healthcare policy and practice through the utilization of research knowledge, skills and practice to improve service delivery and care outcomes. However, this ability to respond to challenges has been less evident in a research context.

Despite their positioning, nurses often lack the research expertise, confidence or engagement required to lead others in the healthcare system. Reasons for this are multifaceted and include a lack of protected time to undertake research training, unstructured career progression pathways to support them, a lack of mentorship and role‐modelling and limited buy in from partner organizations (Henshall, Greenfield, et al., 2020; Henshall, Kozlowska, et al., 2020). This problem is exacerbated in community nursing where there is a lack of clinical academic nurses and sub‐optimal use of the evidence‐base in clinical decision‐making (Bowers, 2018). This is in part due to specific challenges community nurses face in engaging with research; a recent systematic review found that practical barriers in implementing evidence‐based practice in community nursing settings included research implementation challenges, organizational and infrastructure changes, research uncertainty and research being perceived as incompatible with community nursing roles (Mathieson et al., 2018). This combined with a lack of time, heavy patient caseloads, staff shortages and autonomous working practices means that the research component of the community nurse's role is often lacking (Brooke & Mallion, 2016). However, these autonomous working practices make it even more important that community nursing practice is embedded in the evidence base, as community nurses often lack the peer‐to‐peer support in decision‐making that many of their hospital‐based nursing colleagues benefit from. With changes to community nursing practice evolving rapidly, community nurses need support to help them meet the outcomes that matter most to patients and carers (Kenkre et al., 2013).

Many different definitions of community nursing exist, with a lack of clarity around their role. Community nurses are often described as ‘nurses who care for people in their own homes’ (Mathieson et al., 2018). However, in the UK, the boundaries are much more blurred with community nurses acting as the conduit between community, primary and secondary care settings, as well as working closely with social care services and local authorities to ensure the holistic needs of patients are met. Community nurses have long been leaders in promoting this kind of partnership working and the benefit this can bring to patient and service outcomes across healthcare systems have been identified in recent policy documents (NHS, 2021). The National Health Service's (NHS) Long Term Plan sets out its vision for implementing integrated care systems (ICSs) across England by 2021. ICSs promote the dissolution of traditional divisions between hospital and home care, with a view to providing coordinated support across the NHS, social care services, local councils and the voluntary sector (NHS, 2021).

Despite the recognition by the UK government that coordinated working between hospital and community settings is imperative for optimizing patient care outcomes (NHS, 2021), there remains a substantial lack of research investment in community settings, with anecdotal evidence suggesting a disproportionate amount of research funding is directed towards acute hospital care settings. This lack of research funding has had a detrimental effect on community nursing research, adding yet another barrier to community nurses' abilities to engage with the evidence base. These barriers persist at an international level, with an American study reporting on barriers to research nursing utilization in a Magnet community hospital. The study found that barriers included a lack of a supportive environment for research, a lack of research access and availability, a lack of research education and communication and minimal practical application of research (Karkos & Peters, 2006). Furthermore, an Australian study surveying current practice and guideline use in adult cancer pain assessment and management by community nurses found that a range of healthcare system, health professionals and consumer barriers limited access to the best available treatment. The study recommended that an evidence‐based clinical pathway was required to enable community nurses to ensure their patients had access to the best available care (Philips & Lovell, 2015).

Despite efforts by the International Collaboration for Community Health Nursing Research (ICCHNR) to provide support for community nurses on a global level through building an international network of nurse researchers, hosting conferences and funding scholarship awards (ICCHNR, 2021), engagement in evidence‐based practice remains less than satisfactory (Bowers, 2018), with a lack of studies in community nursing (Bowers, 2018). This has potentially devastating consequences for the future of the community nursing profession and its ability to keep up to date with changes across healthcare systems, with resulting implications for the retention and recruitment of staff and the quality and safety of patient care.

To address some of the issues described above, a group of senior nurse and midwife research leaders (SNMRLs) undertook a James Lind Alliance Priority Setting Partnership (JLA PSP) bringing together carers and clinicians in Community Nursing (James Lind Alliance, 2021a), between March 2020 and September 2021, to identify the top 10 evidence uncertainties in this area. This was done to raise the profile of community nursing research and to attract more research funding to this area of healthcare; JLA PSPs focus on issues that are of direct relevance and potential benefit to patients, their carers' and healthcare professionals (James Lind Alliance, 2021b). To work together as a collective with a view to empowering other nurses in the community nursing landscape, concepts and theories from the literature around inclusive leadership were studied and used as a framework with which to conduct the PSP. Table 1 shows how the theoretical concepts underpinning inclusive leadership were reflected on and interpreted by the SNMRLs before being applied to the JLA PSP. Inclusive leadership can be defined as ‘words and deeds by a leader or leaders that indicate an invitation and appreciation for others’ contributions', with inclusive leaders shaping situations where ‘voices are genuinely valued’ (Nembhard & Edmondson, 2006). Furthermore, positive correlations have been found between inclusive leadership styles, innovative work behaviours and psychological empowerment (Javed et al., 2018). Through the provision of a supportive climate and attempts to include others in discussions and decisions where their voices and perspectives might otherwise be absent (Nembhard & Edmondson, 2006), this model of leadership was applied to the JLA PSP in an attempt to empower and inspire others to engage in real‐life issues surrounding community nursing practice.

**TABLE 1:** Reflections on inclusive leadership theory and its application to the JLA process

Theoretical foundations of inclusive leadership	Reflections and interpretation of theory	Examples of how theory was practically applied to JLA process
Acknowledging and valuing everyone's inherent worth	Not seeing people's deficits, but valuing their resources and qualities Sense of worthiness enhances the sense of belonging – this can be stimulated by Inclusive leaders Inclusive leaders value people for their unique identities, perspectives and talents	Engagement from the steering group was appreciated and valued by leaders; members had the freedom to express views/experiences Sense of belonging voiced by the steering group Trusting relationships fostered a psychologically safe working environment SNMRLs from different geographical locations and with different clinical backgrounds, respected and valued diversity of experience
Based on human rights approach	Fosters attitudes and actions to ensure that human rights criteria (availability, accessibility, quality, affordability, acceptability) and principles (non‐discrimination, participation, access to information, accountability and sustainability) are accounted for	PPI members requested using Zoom for meetings as more accessible and fewer problems with connectivity Payment provided for PPIE time Community staff working at all levels welcomed Images used on promotional webpages advocated for diversity
Awareness of interconnectivity	Eco‐systemic awareness emphasizes well‐being Inclusive leadership shifts from seeing individual viewpoint to experiencing the system from the perspective of others, particularly marginalized groups. Goal to co‐sense, co‐inspire, and co‐create an emerging future that values the well‐being of all	Each steering group member used networks to access marginalized groups. Challenging due to COVID as many groups were not meeting. Impact of COVID on staffing and ways of working, reduced team meetings and more lone working, led to fewer opportunities to share surveys and access patients
The role of power	Power is considered the vital energy that drives each person to act and enact change in the direct environment Power finds common ground amongst different interests and builds collective strength	Shared vision amongst steering group PPIE input into promotional materials; steering group reviewed project documents. Final workshop had a diverse representation. Common themes and priorities are determined whilst respecting fellow attendees' perspectives and experiences
Courage to share and take responsibility	Inclusive leaders should invite team members to take up the responsibility to feel part of the process. Empower the team by valuing potential and motivating them to leave their comfort zones.	Sub‐groups required to support survey development Networking responsibilities shared Final workshop preparations and responsibilities shared between steering group

*Note*: Bortini, P., Paci, A., Rise, A., & Rojnik, I. (2018). Inclusive leadership: Theoretical framework. Inclusive leadership. Available online https://inclusiveleadership.eu/ [Accessed 20 Jan 2022].

This paper focuses on the benefits of inclusive leadership in providing a collaborative and committed nurse‐led forum for initiating impactful changes across community nursing, by identifying evidence uncertainties, raising the profile of these uncertainties and driving forward research capacity‐building initiatives in community nursing on a national and international level.

## 2 BACKGROUND

The project sought to address a gap in nursing knowledge by undertaking a JLA PSP in community nursing, to promote and increase engagement amongst community nurses in research at a national level. The JLA is a non‐profit making initiative that brings patients, carers and clinicians together in PSPs. PSPs identify and prioritize evidence uncertainties, or unanswered questions relating to a specific area of healthcare, to ensure that health research funding bodies consider which research questions to prioritize (James Lind Alliance, 2021b).

Mindful of the challenges and barriers to embedding research in the community nursing setting (Bowers, 2018; Karkos & Peters, 2006), four nursing research leaders connected through the National Institute for Health Research's (NIHR) 70@70 Senior Nurse and Midwife Research Leader Programme (NIHR, 2019). The SNMRLs articulated a shared commitment to collectively working together during the SNMRL programme on this project to identify community nursing research priorities and to empower community nurses to explore and engage with research that is directly related to their practice and patient care outcomes. The 70@70 SNMRL Programme was established in 2019 as a 3‐year programme which aimed to strengthen the research voice and influence of nurses and midwives in health and social care settings in England, with a view to building research capacity and capability amongst nurses and midwives (Henshall, Greenfield, et al., 2020). SNMRL cohort members were provided with 2 days' protected time each week as part of the 70@70 Programme to drive forward research innovations and initiatives to generate, lead and support research activity, as well as informing research priorities at a local, regional and national level (Henshall, Greenfield, et al., 2020).

The community nursing PSP's aim was to define the top 10 evidence uncertainties relating to community nursing through a shared, inclusive partnership with key stakeholders including patients, carers, community nurses and other community‐based healthcare workers. The breadth and diversity of community nursing roles meant that the JLA PSP's scope needed to be well‐defined. As such, the PSP's scope set out to identify evidence uncertainties for community nursing in England, with a focus on the provision of nursing care to adults in their own homes, in community clinics or in residential homes. Community nursing encompasses a diverse range of nurses and support staff working in the community including district nurses, intermediate care nurses, community matrons and hospital at home nurses. Community nurses have knowledge and experience in supporting people with multi‐morbidities, acute illness, chronic and long‐term conditions, such as heart failure, chronic obstructive pulmonary disease, multiple sclerosis, Parkinson's disease, cancer and diabetes. They focus on preventative, coordinated care to avoid hospital admissions and facilitate self‐management at home (Maybin et al., 2016). This support facilitates improvements in quality of life, promotes independence and offers a patient‐centred, supportive and appropriately applied service from diagnosis to end‐of‐life care (Department of Health, 2005). The evolving complexity of patient care requirements means community nurses continually respond and adapt to meet the needs of local populations (Maybin et al., 2016).

To ensure PSP inclusivity, stakeholders' views were collated at a national level through the dissemination of two surveys. Survey findings identified questions that were prioritized by a steering group, with members including Chief Nurses, community nurses, patients and carers. The first steering group meeting took place in September 2020, with PSP surveys conducted between December 2020 and July 2021. Separate surveys were sent to community‐based health care professionals and to patients and carers who had utilized community nursing services. The healthcare professional survey contained questions about what needed addressing in community nursing settings. The patient and carer survey asked respondents to identify what community nursing teams did well and what could be improved. Over 700 responses to the initial surveys were received, despite pressures from COVID‐19. Responses were grouped together and collated until 40 overarching questions were developed. The 40 questions related to a range of community nursing and patient‐focused topics. Topics included caring for the complex needs of patients; promoting health and self‐management strategies for patients and their families; optimizing integrated working practices, improving community nurses' staffing ratios; enhancing the wellbeing of community nursing staff; and retention and recruitment issues. The questions formed the basis for the second survey, which was sent to community healthcare professionals, patients and carers, who ranked these 40 questions in priority order. A final workshop, based on the top 18 prioritized questions that were ranked by survey two respondents, took place in September 2021 to agree on the top 10 priority questions or evidence uncertainties. The PSP process is outlined in Table 2. Funding was provided by the NIHR Applied Research Collaboration, which supports applied health research innovations that make a difference in patient care outcomes.

**TABLE 2:** An outline of the steps involved in the James Lind Alliance priority setting process

The priority setting partnership process
Create a steering group with equal representation of patients, carers and clinicians and strong links to relevant partner organizations; and raise awareness of the project to maximize support and participation.
Gather evidence of uncertainties by asking patients, carers and clinicians to respond to a survey asking what questions they have for research, and by searching existing literature to identify gaps.
Refining questions and uncertainties – the steering groups work with a JLA information specialist to create a long list of summary questions from the survey responses.
Evidence checking—the long list of summary questions is checked against the existing research evidence to ensure that they have not previously been answered by research.
Interim prioritization – through consensus, the steering group prioritizes the identified uncertainties by asking patients, carers and clinicians to complete a second survey to rank the research questions.
Final prioritization – this is generally a one‐day workshop facilitated by the JLA and with input from the steering group; up to 30 patients, carers and clinicians will participate in discussion and ranking to determine the top 10 questions for research.
Publish and promote Top 10 research priorities ‐ the Top 10 is announced and published and a publication and promotion plan is implemented to disseminate the results and influence researchers.

*Note*: James Lind Alliance. (2021). JLA Guidebook. Retreived from https://www.jla.nihr.ac.uk/jla‐guidebook/ [Accessed 20 Jul 2021].

This discussion paper is based on shared reflections of four SNMRLs who undertook the PSP in community nursing; learning points are supported throughout the paper by relevant literature and theory. The SNMRLs were situated in geographically diverse locations across England and had different professional nursing backgrounds; they were well placed to undertake the PSP using an inclusive leadership model (Bortini et al., 2018), as they could access a range of stakeholders with varying demographic characteristics across the population. This enhanced representation and ensured that the top 10 evidence uncertainties identified through the JLA PSP process were informed through a wide variety of viewpoints, contexts and perspectives (James Lind Alliance, 2021b). Their inclusive leadership style was evident throughout the JLA PSP, with every effort made to seek out the views of patients, carers, community nurses and other healthcare workers who had an experience in the community working across a range of geographical and healthcare settings (Table 1). This collaborative model meant the SNMRLs could share ideas, develop extended networks and reach geographical locations across England that would not otherwise have been possible. This paper considers how four SNMRLs adopted an inclusive leadership approach to support and enable the delivery of a priority‐setting partnership in community nursing, through a collaborative and committed nurse‐led forum. The process of gathering evidence uncertainties, through national surveys and delivering a final inclusive workshop to prioritize them, is detailed.

## 3 DISCUSSION

### 3.1 Data sources

This discussion paper is based on shared reflections, through extensive note‐taking after meetings and frequent team discussions (Gibbs, 1988), supported by relevant literature and inclusive leadership theory (Bortini et al., 2018). The paper draws on four main areas that emerged relating to the PSP. These were collective leadership, stakeholder engagement, diversity and inclusivity and the impact of COVID‐19.

### 3.2 Collective leadership

Many collective benefits emerged from the PSP, not least the ability to influence and lead through collaboration. As a core team of four nursing leaders, the SNMRLs drew on individual strengths, skill sets and reflections to enable the diversity of thinking, ideas and perspectives. Rather than one individual leading the PSP, the collaborative, inclusive nature of the SNMRL team meant that a supportive environment for sharing plans, strategies and processes was cultured. This inclusive leadership style enabled the PSP to have a collective impact in terms of the people, organizations and systems it infiltrated and influenced. Throughout, the PSP engagement was sought at a grass roots level locally and at a national level, to influence policy and practice in healthcare systems. This ability to engage at all levels was facilitated through the 70@70 platform (Henshall, Greenfield, et al., 2020) which allowed existing links in local trusts to be capitalized on, whilst providing collective influence through the national profile of the programme. As the 70@70 programme was affiliated with England's National Institute for Health Research, this made it easier for the JLA PSP to gain traction and for its aim and purpose to be articulated by leaders at national meetings where policy and funding decisions were considered. This led to buy in and positive acknowledgement from external stakeholders involved in research policy, practice, design and delivery, which generated momentum for how the outputs from the PSP could be embedded and sustained nationally. Funding opportunities and decisions to deliver on the top 10 evidence uncertainties were also generated and secured through this approach.

### 3.3 Stakeholder engagement

Increasingly, there is a requirement in research funding applications for patients and the public to be an integral part of the research design, delivery and dissemination process. Lack of involvement and engagement from these individuals can have a negative impact on successful outcomes, as an application's perceived value and credibility are undermined (Horrocks et al., 2018). This meant that in the JLA PSP, the research priorities of healthcare professionals' patients' and the public needed to be identified, listened to and heard, to appreciate the true complexities and context of the area under investigation (Schot et al., 2019). The JLA PSP process epitomizes this concept through its collaborative nature, working with patient and clinical communities, without imposing a top‐down, inflexible set of rules. Whilst the JLA PSP leaders' imposed structure and process to the project, the stakeholders contributed knowledge, experience, insights, networks and personal and professional investments in future research, in and across their spheres of interest. This led to a sense of empowerment as the shared research priorities generated from the PSP belonged to everyone involved (James Lind Alliance, 2021b).

Throughout the PSP, it was anticipated that community nurses at a local and national level would engage with, enhance and expand their involvement in research and start to critically analyse their own clinical practise to ensure its alignment with up‐to‐date evidence. The collaborative, inclusive approach generated by the SNMRLs was adopted by local and national nursing research leaders and was a crucial component in promoting and supporting active involvement in the PSP from the nursing community. Research champions for the PSP were linked to local hospitals and this led to them sharing details of the PSP at strategic and operational levels. This resulted in the development of an engaged community with a common purpose of progressing and enabling involvement in community nursing research.

To ensure that the contribution of community‐based healthcare professionals was heard throughout the PSP, each SNMRL recruited community and Chief Nurses from their own organizations to join the steering group. It was important to reach the Chief Nurses to achieve full engagement and commitment across the different organizations, whilst gathering perspectives from community nurses resulted in valuable insights about what the frontline issues and challenges facing them were. This widespread representation from healthcare professionals, patients and carers, engendered confidence that the views of people living and working in different communities with varying socio‐economic needs and healthcare priorities were being heard. The theoretical underpinnings of inclusive leadership provided a lens for the team to view leadership and change processes as emerging through networked forms of communication, relationships and influence (Nembhard & Edmondson, 2006). This was achieved by understanding the views and needs of the people involved in the process and by actively drawing on the diversity and richness of different perspectives, backgrounds and experiences (Amin et al., 2018). This enabled the SNMRLs to become more adept at including the wider stakeholder group in PSP discussions and decision‐making and to facilitate a supportive environment to encourage sharing of opinions and perspectives (Nembhard & Edmondson, 2006) (Table 1).

### 3.4 Diversity and inclusivity

The four SNMRLs held a broad variety of roles including Lead Community Research Nurse, Research and Development Manager, Clinical Academic Nurse and Deputy Head of Nursing. They also spanned different geographical locations from the North West to the Midlands, South East and South England, working in a range of rural and urban communities. The hospitals they were based at included acute and community health, mental and community health and stand‐alone community settings. A significant benefit of this professional role diversity was that it generated multiple ideas and perspectives that related to the varying organizational pressures and priorities of different organizations and communities. This diversity in leadership is cited as beneficial for delivering a more inclusive leadership approach due to the dissipation of interpersonal tensions that are more likely to occur in more homogenous teams, disrupting the integration of opinions as a result (van Knippenberg & van Ginkel, 2021). The professional role diversity of the SNMRLs also enabled them to access and utilize a wide range of networks at a local, regional and national level including community nurses in local teams, care home residents, patients living in the community, local chaplains, research and development teams, national community nursing bodies, healthcare organizations, policy makers and professional bodies. Access to a diverse stakeholder group enabled the SNMRLs to stay close to issues that really mattered to patients, carers and frontline nurses, whilst increasing opportunities to leverage power and influence policy and practice at a national level and to collaborate through new connections and mutually beneficial interactions across the healthcare system.

Increased recognition is being paid to ensuring that under‐served groups should be represented on research boards and committees, such as funding panels and ethics committees, where any barriers to participation should be identified and resolved as a means of promoting inclusivity (Witham et al., 2020). In terms of the PSP, every effort was taken to ensure that a representative mix of steering group members and survey responders with different genders, ethnicities and geographical locations was obtained; however, this was not without its challenges. The steering group meetings included representation from a variety of ethnicities and cultures, which led to meaningful discussions relating to survey development. For example, the survey wording was altered to reflect the fact that in some cultures, family members do not identify as ‘carers’, but maintain their identity as a husband, wife or family member (Hughes et al., 2013). These insights supported the group to develop a survey that was as inclusive as possible. This collaborative nature of the group also meant that relationships and confidence grew over time, with a growing focus on the purpose of the team's shared objectives.

**FIGURE 1:**
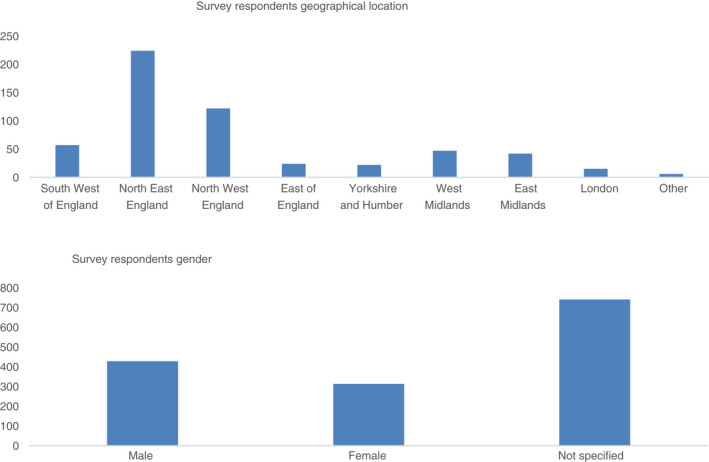
Demographics of James Lind Alliance priority setting partnership in community nursing survey responders.

In terms of the survey, contributions from patients and carers were continuously monitored to promote inclusivity and diversity of the responders (Figure 1). During the dissemination of the first survey, it became clear that certain sectors of the population were underrepresented. However, with regard to gender, this was not the case. The NMC register to practice, records more women (89%) than men (11%) on its nursing register (NMC, 2021). This is in line with our survey findings which recorded 92% of responses from women and 8% from men, largely representative of the nursing workforce. Geographically, there was representation from all areas of England, although 62% of all respondents came from the North. Most survey respondents were people who identified as being of White ethnicity (94%). The remaining 6% were from a range of ethnically diverse backgrounds including Black, African, Caribbean, Black British, Asian British (1.7%), Asian (1.4%), Mixed, multiple ethnic groups (0.8%) and other (0.5%) with the remaining 1.6% preferring not to say. Diversity was monitored throughout the PSP process, however, due to the challenges of non‐face‐to‐face contact and an inability to target specific religious groups during the COVID‐19 pandemic, we were unable to achieve the level of diversity that we would have liked. Efforts to rectify this included snowball sampling, utilizing personal contacts to increase diversity at the start of the process and the identification of community gatekeepers to explain the value and purpose of the research to hard‐to‐reach communities (Lee, 2005). Other strategies included utilizing social media platforms and targeting communications to key stakeholder groups, as well as connecting with individuals via email as a means of building the PSP network and staying connected virtually. Despite these outreach initiatives, limited progress was made. This may be partly due to the COVID‐19 pandemic which meant that many community nurses were under enormous workplace pressures and were unable to prioritize the dissemination of the surveys to community groups.

### 3.5 The impact of COVID‐19

Despite the challenges faced, with the UK having one of the highest rates of infection globally at certain timepoints during the pandemic (Anderson et al., 2020) and many nurses being redeployed to frontline care, the PSP demonstrated the commitment felt by community nurses, patients and carers to improve the research standing of the profession. The imposed transition from face‐to‐face meetings to virtual, online platforms led to considerations as to how regularly the leadership team and the steering group should communicate, as well as how to best ensure that the voices of patients and carers who could not access online platforms could be heard. Although coordinating the project virtually had drawbacks in terms of not being able to connect in a face‐to‐face setting, there were advantages, including a reduction in travel time for all stakeholders, reduced project costs and increased productivity and efficiency through making better use of shorter online meetings. The SNMRLs promoted a non‐hierarchical culture throughout the process, stipulating that every individual's voice and perspective was equal and should be treated with respect. This required experienced facilitation to ensure that everyone's voice was listened to and considered (James Lind Alliance, 2021b). Equality amongst the group was valued by all members and led to richer and more meaningful discussions taking place. It also helped to reduce potential anxieties about speaking up that can occur in healthcare settings when senior team members can monopolize meeting agendas.

During the PSP, the SNMRLs were aware that a sector of the patient and carer population might not have easy access to the JLA PSP online and paper surveys, leading to their potential exclusion from the process. An ONS survey in 2020 found that although 96% of UK households had access to the internet, issues of age and disability remained key barriers to adoption. Only 67% of those over 65 s classified themselves as daily or almost daily internet users and people considered to be disabled reported lower than average internet use when compared with the general population (Office for National Statistics, 2020). This lack of online access is problematic for people from lower socio‐demographic backgrounds, as they may have greater health issues and need more healthcare professional support. This need for support is magnified in the community setting, where in the UK, it is estimated that 51% of people aged over 75 years live alone (NHS Choices, 2015) and a visit from a community nurse may be their only point of social interaction. To overcome these barriers during the COVID‐19 pandemic, the SNMRLs tried to ensure that PSP surveys were made available to patients and carers who were not easily able to access them. This proved difficult due to the social restrictions imposed by COVID‐19, as leaving paper surveys in community settings such as general practices, churches or community centres was not permitted. However, the SNMRLs who visited patients in their homes provided paper surveys and invited individuals to complete them with assistance. This allowed surveys to be completed by a small subsample of patients and carers, including the elderly and frail. However, only a small number of surveys were collected using this method due to difficulties including the ill health of patients and the time constraints of the SNMRLs. Due to the ever‐changing challenges relating to COVID‐19 that community nurses were facing, the SNMRL team decided not to ask community nurses to take a role in disseminating the surveys or supporting patients and carers to complete them. However, the community nurses were asked to complete the surveys themselves and to promote them across their organizations, via their communications teams and other methods, including word of mouth.

### 3.6 Implications for nursing

An international study investigating how nursing leaders promote evidence‐based practice found that the involvement of patients was lacking throughout this process (Kitson et al., 2020), highlighting the importance of an inclusive approach to ensure that the voices of all relevant parties are listened to and considered. The PSP was careful to engage with patients, carers and community nurses at all levels and has provided a catalyst for developing rigorous evidence in community nursing practice. The collaborative opportunities, networks and connections that have developed with patients, carers, nursing leaders, policy makers and healthcare colleagues across the country have allowed new relationships to be developed, thereby raising the profile of the PSP and increasing the likelihood of sustained impact. This can lead to widespread benefits for nursing research, practice and education, and most importantly, for patients who will be in receipt of community nursing care in the future.

Collective buy in and engagement with the JLA PSP from national leaders in areas such as policy, education, funding and commissioning has resulted in a commitment that the evidence uncertainties identified will be funded and focused on in the future. However, in addition to funding the evidence uncertainties, more widespread action is required to ensure the legacy of the PSP is long‐lasting and influential. The creation of research strategies developed to support community nursing research, such as the Welsh Community Nursing Research Strategy (Kenkre et al., 2013), is one way to facilitate the embedding of evidence in community nursing, by supporting nurses with the right research infrastructure and encouraging them to play an active part in developing community nursing research and evidence‐based practice (Kenkre et al., 2013).

National and international bodies, such as the Queens Nursing Institute and the ICCHNR, align with the ethos of the community nursing PSP as they support the importance of utilizing evidence to inform practice (ICCHNR, 2021; The Queens Nursing Institute, 2021). This is important in terms of ensuring that the findings generated by the PSP are implemented in national and international policies linking to nursing practice, education and research. Additionally, in 2018 the Care Quality Commission, which regulates the NHS, formally recognized clinical research activity in the NHS as a key component of best patient care (Care Quality Commission, 2021). This is significant in terms of positioning, as research is now recognized as a key part of patient care in the NHS Constitution. As such, NHS managers will be seeking to increase levels of research activity in their clinical areas and outputs from the PSP can be used as the basis for ensuring that research priorities are examined and embedded in frontline community settings and that the projects undertaken are meaningful and add value to patient care pathways.

## 4 CONCLUSION

The 70@70 SNMRL programme facilitated a shared platform for nurses working across different healthcare boundaries, with different healthcare priorities, to raise the profile of community nursing research, with international implications for nursing practice. The programme promoted inclusive leadership and empowered SNMRLs to initiate capacity and capability‐building strategies that were made possible due to the profile and connectivity resulting from it. The diversity in leadership, organizational representation, networking and geographical scope of the PSP provided an opportunity to benefit those working and living in the community in the long term.

Revitalizing an area of practice to address the lack of evidence underpinning the activities of a profession requires a truly collaborative and inclusive effort to be effective. Seen through the lens of inclusive leadership, contributors to the process were encouraged to raise the voice of less represented views and to challenge their own biases and preferences. The value of applying the theoretical principles of inclusive leadership to practice (Table 1), and the national collaborative effort this entailed, led to the creation of a national network of nurses, patients and carers that developed research priorities to change the way community nurses' practice. An inclusive leadership approach was effective in generating momentum in community nursing research and transcended the outcomes of the PSP itself. The sustained engagement that has been harnessed from the process can be applied across practice, research and education, with a focus on discovering the best ways of translating and applying research findings into practice and vice‐versa. This model of inclusive leadership can be applied to other areas of nursing outside of community settings and across international nursing contexts and cultures, by prioritizing shared decision‐making and empowering others to develop tangible outputs that can change practice for the better.

Through its inclusive approach the JLA PSP has ensured that its legacy goes further than solely ascertaining funding to deliver research on the top 10 evidence uncertainties; it has also increased engagement, created capacity and capability‐building initiatives and raised the profile of community nursing research. This is essential to ensure that research becomes a core part of the practice and that community nurses feel empowered to lead changes to practice through questioning the evidence base. Continued, sustained engagement and commitment is required to influence funders of research, integrate research priorities into community nursing research, education and practice and drive forward changes to commissioning and service delivery as a means of optimizing patient care.

## FUNDING INFORMATION

This study was funded by the National Institute for Health Research Applied Research Collaboration.

## CONFLICT OF INTEREST

No conflict of interest has been declared by the authors.

### PEER REVIEW

The peer review history for this article is available at https://publons.com/publon/10.1111/jan.15342.

## Data Availability

The data that support the findings of this study are available from the corresponding author upon reasonable request.
